# Research progress of deubiquitinating enzymes in cerebral ischemia-reperfusion injury

**DOI:** 10.3389/fnagi.2025.1588920

**Published:** 2025-06-02

**Authors:** XiaoHong Qin, JiangRui Zhu, HaoRan Lu, MaoRui Yi, ZiLong Zhao, WenFei Zhang, Jing Cheng

**Affiliations:** ^1^Department of Neurosurgery, Renmin Hospital of Wuhan University, Wuhan, China; ^2^Central Laboratory, Renmin Hospital of Wuhan University, Wuhan, China

**Keywords:** cerebral ischemia-reperfusion injury (CIRI), deubiquitinases (DUBs), ischemic stroke, neuroinflammation, oxidative stress, programmed cell death (PCD), ubiquitin-proteasome system, targeted therapy

## Abstract

Cerebral ischemia-reperfusion injury (CIRI) is a critical pathological process driving neurological deterioration following ischemic stroke, involving multifaceted mechanisms such as inflammatory cascades, oxidative stress, and programmed cell death (PCD). Deubiquitinases (DUBs), as key regulators of the ubiquitin-proteasome system, dynamically modulate protein stability, signal transduction, and subcellular localization through editing the ubiquitin code, exhibiting dual roles in CIRI—both as drivers of pathogenesis and potential therapeutic targets. This review systematically elucidates the core regulatory mechanisms of DUBs in CIRI: (i) suppression of neuroinflammation via modulation of NLRP6/NF-κB pathways; (ii) mitigation of oxidative stress through the KEAP1-NRF2 axis and mitochondrial quality control; and (iii) neuroprotection by intercepting necroptosis, ferroptosis, and other PCD pathways. We further reveal that CIRI disrupts DUBs functionality through a tripartite mechanism—transcriptional dysregulation, catalytic inactivation, and subcellular mislocalization—transforming DUBs from guardians of homeostasis into mediators of injury. Consequently, DUBs-targeted strategies, including small-molecule inhibitors (e.g., IU1, Vialinin A), genetic editing approaches (e.g., BRCC3 silencing, A20 overexpression), and exosome-based delivery systems (e.g., the KLF3-AS1/miR-206/USP22 axis), demonstrate significant neuroprotective potential. However, challenges persist, such as substrate specificity, ubiquitin chain-type dependency, and barriers to clinical translation. Future research must integrate multi-omics technologies, develop brain-targeted delivery platforms, and explore synergistic effects of DUBs modulation with existing therapies to advance precision medicine in stroke treatment.

## 1 Introduction

Stroke is defined as a disease that causes damage to the blood vessels of the brain due to a variety of etiologies, resulting in the necrosis of brain cells and tissues. It ranks as the third leading cause of death in our country, following heart disease and cancer. As of 2023, the incidence of stroke in China stands at ~246.8 per 100,000 population, with a mortality rate of around 149.5 per 100,000. The economic burden of stroke has risen

substantially, with average annual treatment costs exceeding 100,000 yuan (Tu and Wang, 2023). Ischemic stroke (IS), accounting for 75–90% of cerebrovascular events (Jing et al., 2023), is characterized by abrupt blood flow cessation leading to neuronal necrosis within minutes. While reperfusion therapies (thrombolysis/thrombectomy) remain cornerstone treatments, cerebral ischemia-reperfusion injury (CIRI) paradoxically exacerbates damage through inflammatory cascades, oxidative stress, and programmed cell death (PCD; Cheng et al., 2021; Hecht et al., 2021; Campbell and Khatri, 2020).

Ubiquitin is a highly conserved 76-amino acid protein that can covalently conjugate to substrate proteins (a process termed ubiquitination; Hershko and Ciechanover, 1998). Ubiquitin molecules can form functionally distinct chains through linkages via any of their seven lysine residues (K6, K11, K27, K29, K33, K48, K63) or the N-terminal methionine. Among these, K48-linked polyubiquitin chains primarily target proteins for proteasomal degradation, whereas K63-linked chains predominantly regulate signal transduction and protein interactions without directly promoting degradation (Komander and Rape, 2012). Ubiquitin modification, a highly conserved regulatory mechanism across eukaryotes, plays a crucial role in controlling protein stability, activity, and subcellular localization through diverse cellular processes (Muratani and Tansey, 2003; Kim et al., 2011). Deubiquitinases (DUBs) regulate protein degradation and signal transduction by removing ubiquitin modifications from proteins. These enzymes are classified into two main categories: cysteine proteases and metalloproteinases (Amerik and Hochstrasser, 2004). The former can be categorized into several distinct protein families based on sequence conservation and domain structure. These include the ubiquitin C-terminal hydrolase (UCH), ubiquitin-specific protease (USP), ovarian tumor protease (OTU), and Machado-Josephin disease protease (MJD) families. Additionally, the K48 polyubiquitin-specific MINDY domain family (MINDY) and the recently identified zinc fingers with UFM1-specific peptidase (ZUP1) domains represent emerging classes of DUBs (Li and Reverter, 2021). The latter consists of zinc-dependent enzymes with JAB1/MPN/MOV34 (JAMM/MPN+) domains.

Protein homeostasis collapse represents a hallmark of CIRI, wherein dysregulated ubiquitination drives pathological progression (Liu et al., 2020; Hochrainer, 2018). As critical regulators of the ubiquitin signaling network, DUBs orchestrate essential cellular processes including: ubiquitin recycling and proteostasis (Park and Ryu, 2014; Guo et al., 2024; Vostal et al., 2025), membrane trafficking and receptor regulation (Clague and Urbé, 2017; Cheng and Guggino, 2013), DNA repair and translation (Foot et al., 2017; Kee and Huang, 2016), cell cycle control and autophagy (Park et al., 2019; Jacomin et al., 2018), and mitochondrial quality control under homeostatic conditions (Bingol et al., 2014; Escobar-Henriques, 2014; Marcassa et al., 2018; Gersch et al., 2017). Notably, recent breakthrough research reveals that CIRI subverts DUBs functionality through three synergistic mechanisms: (1) transcriptional chaos, characterized by polarized DUBs dysregulation in ischemic cores (e.g., BRCC3 upsurge vs. USP16 downregulation; Huang X. et al., 2021; Tong et al., 2023); (2) catalytic crippling via ROS-mediated oxidation of essential catalytic cysteine residues (Forman et al., 2017); and (3) spatial disarray exemplified by USP30 displacement from mitochondrial outer membranes (Chen et al., 2021). This pathogenic trilogy transforms DUBs from vigilant guardians of proteostasis into active drivers of neuronal injury—a fundamental paradigm shift underscoring their dual nature as both instigators of CIRI pathogenesis and promising therapeutic targets for intervention.

This review systematically elucidates the regulatory roles of DUBs in CIRI through three pivotal pathological axes: (i) orchestration of inflammatory response and neuroprotection, (ii) modulation of oxidative stress defense systems, and (iii) intervention of PCD pathways. By comprehensively dissecting the molecular mechanisms of key DUBs (e.g., USP16, A20, OTUD1) and their spatiotemporal dynamics in CIRI, we not only advance the mechanistic understanding of stroke pathogenesis but also provide a conceptual framework for developing DUBs-targeted therapeutic strategies.

## 2 General functions of DUBs in cellular homeostasis

### 2.1 Ubiquitin recycling and proteostasis

DUBs play a crucial role in maintaining the cellular ubiquitin pool by recycling and generating free ubiquitin molecules, which are essential for protein degradation and signaling pathways (Park and Ryu, 2014). Protein ubiquitination and deubiquitination are important forms of post-translational modification. Ubiquitination involves the regulation of protein stability or activity through ubiquitin ligases, which attach ubiquitin chains to proteins, marking them for degradation. Deubiquitination, a crucial post-translational modification process, functions by either modifying ubiquitin molecules and chains or removing polyubiquitin chains from target proteins, thereby counteracting protein ubiquitination and subsequent degradation (Guo et al., 2024; Vostal et al., 2025).

### 2.2 Membrane trafficking and receptor regulation

At the plasma membrane, ubiquitination serves as a critical regulatory mechanism for protein endocytosis, a cellular process whereby vesicles form to internalize extracellular materials and cell-surface proteins. This regulation can be achieved through the ubiquitination of either cargo proteins directly or their associated adaptor proteins, thereby modulating both the efficiency and specificity of the endocytic pathway (Clague and Urbé, 2017). The ubiquitinated cargo undergoes transport to endosomes, lysosomes, or the endoplasmic reticulum for processing or degradation (Cheng and Guggino, 2013; Fujita et al., 2013; Haglund et al., 2003; Piper et al., 2014). In these organelles, DUBs can remove ubiquitin tags from cargo proteins, preventing their degradation and enabling their recycling to cellular surfaces or other destinations (Balut et al., 2011; Clague and Urbé, 2006). Ubiquitination plays a crucial role in regulating cell surface receptor dynamics through its dual control of receptor endocytosis and signal transduction. For example, epidermal growth factor receptor (EGFR), upon ligand binding, is ubiquitinated and internalized into endosomes, thereby terminating its signaling. USP8 prevents EGFR degradation by removing ubiquitin tags, thus facilitating EGFR recycling to the cell surface and sustaining signal duration (Berlin et al., 2010; Hurley and Stenmark, 2011).

### 2.3 DNA repair and translation

DUBs regulate DNA damage repair mechanisms to ensure their availability during cellular stress and damage responses (Foot et al., 2017; Kee and Huang, 2016). Under normal conditions, phosphorylated USP7 stabilizes MDM2, which then promotes p53 degradation through ubiquitination (Sarkari et al., 2010; Sheng et al., 2006). Upon cellular stress, USP7 dephosphorylation causes murine double minute 2 (MDM2) destabilization, resulting in p53 accumulation (Khoronenkova et al., 2012). DUBs also regulate protein translation by modifying ribosomal ubiquitination (Kapadia and Gartenhaus, 2019). DUBs play a crucial role in regulating global protein synthesis. A newly discovered regulatory mechanism in yeast, called redox control of translation by ubiquitin (RTU), involves ribosomal DUBs (Dougherty et al., 2020). Ubp2 (a ubiquitinating enzyme) at the center of the pathway is reversibly inhibited by reactive oxygen species (ROS) during oxidative stress in RTU. This inhibition results in the accumulation of K63-linked polyubiquitin chains on the ribosome, catalyzed by ubiquitin-conjugating enzymes Rad6 and ubiquitin-protein ligase (E3) Bre1, which subsequently blocks translation elongation (Simões et al., 2022).

### 2.4 Cell cycle control and autophagy

Not only that, DUBs also play crucial roles in mitosis, as exemplified by USP7, which controls G1-S and G2-M checkpoints through claspin deubiquitination. USP16 controls chromosome segregation and alignment through its deubiquitinating activity on histones H2A and PLK1, thereby regulating G0 and early M phase. Independently of the spindle assembly checkpoint, USP28 modulates the G1-S checkpoint by deubiquitinating p53 (Park et al., 2019).

In addition, DUBs can also participate in the regulation of autophagy. For example, during macroautophagy, double-membrane phagosomes and autophagosomes initially form within the cytoplasm. The mammalian target of rapamycin (mTOR) primarily regulates phagosome formation, while DEP domain-containing mTOR-interacting protein (DEPTOR) acts as its negative regulator by inhibiting mTOR kinase activity. Under normal conditions, DEPTOR undergoes continuous degradation through the ubiquitin-proteasome system. During nutrient deprivation, OTUB1 prevents DEPTOR degradation, which is essential for mTOR inactivation (Jacomin et al., 2018).

### 2.5 Mitochondrial quality control under homeostatic conditions

USP30, a predominantly mitochondria-localized deubiquitinase anchored to the outer membrane, plays pivotal roles in homeostatic mitochondrial quality control through the following mechanisms. (1) Negative Regulation of PINK1/Parkin-Mediated Mitophagy: USP30 counteracts ubiquitination of outer membrane proteins (e.g., TOM20, MFN2), thereby suppressing excessive mitophagy initiation (Bingol et al., 2014). (2) Modulation of Mitochondrial Dynamics: By deubiquitinating the fusion GTPase MFN1, USP30 preserves mitochondrial fusion capacity, ensuring network homeostasis (Escobar-Henriques, 2014; Yue et al., 2014). (3) Oxidative Stress Defense: USP30 stabilizes mitochondrial antioxidant proteins (e.g., SOD2), mitigating ROS accumulation (Marcassa et al., 2018). (4) Energy Metabolism Maintenance: Through deubiquitination of electron transport chain subunits (e.g., NDUFB10), USP30 safeguards respiratory chain integrity and oxidative phosphorylation efficiency (Gersch et al., 2017).

While these general functions maintain cellular homeostasis, DUBs exhibit context-specific roles under pathological conditions such as CIRI, as discussed below.

## 3 Regulatory mechanisms of DUBs in IS: from homeostasis to pathology

### 3.1 Physiological regulation of DUBs activity

The activity of DUBs is tightly controlled through three principal regulatory strata: post-translational modifications, protein-protein interactions, and transcriptional networks, collectively maintaining cellular homeostasis. At the post-translational level, phosphorylation emerges as a dominant regulatory switch; USP14, for example, acquires full catalytic competence only through dual regulation by proteasomal association and AKT-dependent phosphorylation (Wang et al., 2021), whereas cylindromatosis (CYLD, a USP-family deubiquitinase) undergoes functional silencing via Ser418 phosphorylation (Hutti et al., 2009). The regulatory landscape extends to redox control, where ROS induce transient catalytic inactivation of cysteine-dependent DUBs (including USP and OTU family members) through reversible oxidation of the active-site cysteine to disulfide states (Clague, 2013; Cotto-Rios et al., 2012; Kulathu et al., 2013; Lee et al., 2013). Structural orchestration plays an equally critical role—USP14 and UCHL5 achieve functional activation exclusively through incorporation into the 19S proteasomal regulatory complex (de Poot et al., 2017), while WDR48/WDR20 allosterically activates USP46 via precise β-propeller domain engagement (Cohn et al., 2009; Kee et al., 2010; Yin et al., 2015). Complementing these mechanisms, an intricate layer of transcriptional governance exists, featuring miRNA-directed mRNA degradation (notably miR-124-mediated USP14 suppression) and lncRNA-operated competitive endogenous RNA (ceRNA) networks (exemplified by KLF3-AS1's sequestration of miR-206 to derepress USP22 expression; Song et al., 2019; Xie et al., 2023).

### 3.2 Dysregulation of DUBs in CIRI

CIRI disrupts DUBs regulatory networks through three principal mechanisms: (i) Transcriptional dysregulation, where ischemic stress induces BRCA1-BRCA2-containing complex subunit 3 (BRCC3, a JAMN domain-containing Zn^2+^metalloprotease deubiquitinating enzyme) upregulation to activate the NOD-like receptor family pyrin domain containing 6 (NLRP6) inflammasome (Huang X. et al., 2021), while nuclear factor erythroid 2-related factor 2 (NRF2)-mediated negative feedback suppresses USP16 expression, impairing Kelch-like ECH-associated protein 1 (KEAP1) degradation (Tong et al., 2023); (ii) Catalytic impairment, as ROS accumulation causes irreversible oxidation of UCH-family DUBs (Forman et al., 2017; Kahles et al., 2021; Defelipe et al., 2015; Devarie-Baez et al., 2016), and energy depletion reduces AKT-mediated activating phosphorylation of USP14 (Wang et al., 2021); (iii) Subcellular mislocalization, exemplified by USP30 dissociation from mitochondrial membranes, leading to pathological mitofusin 2 (MFN2) hyperubiquitination and mitochondrial fragmentation. These coordinated perturbations collectively compromise the homeostatic regulatory functions of DUBs (Chen et al., 2021).

As reviewed, the functional impact of DUBs in CIRI is governed by three fundamental determinants: substrate specificity, ubiquitin chain selectivity, and spatiotemporal regulation. Under physiological conditions, DUB activity is precisely constrained within homeostatic boundaries. However, CIRI induces three distinct pathological cascades: (i) ROS-mediated oxidation inactivates cysteine-dependent DUBs (e.g., UCHL1); (ii) bioenergetic failure impairs kinase-dependent DUB activation (e.g., the AKT-USP14 axis); and (iii) transcriptional reprogramming alters DUB expression profiles (e.g., NF-κB-driven BRCC3 upregulation). This triad of disturbances transforms DUBs from homeostatic guardians to disease modifiers, capable of either exacerbating injury (as demonstrated by USP16/KEAP1-mediated NRF2 suppression) or facilitating recovery (exemplified by OTUD1-RIP2's anti-inflammatory activity). Given their pleiotropic regulation of core CIRI pathways—including inflammatory responses, oxidative stress, and programmed cell death—the mechanistic dissection of DUB networks warrants systematic investigation.

## 4 Mechanisms of DUBs in CIRI

Recent studies have shown that DUBs play an important regulatory role in CIRI, affecting the survival, death, and repair of neurons (Liu et al., 2020; Hochrainer, 2018; Chen et al., 2020). DUBs modulate the inflammatory response by suppressing the release of inflammatory mediators, thereby mitigating cellular damage. Additionally, these enzymes regulate oxidative stress responses following CIRI by reducing free radical production, thus protecting cells from oxidative damage. Furthermore, DUBs contribute to cellular protection through their involvement in PCD, specifically by regulating processes such as ferroptosis and mitophagy (Zhang et al., 2023; Marques et al., 2019; Lu et al., 2021; Jayaraj et al., 2019).

### 4.1 Inflammatory responses modulation and neuroprotection

The inflammatory response in CIRI is a complex process involving the activation and regulation of multiple signaling pathways. The NLRP6 inflammasome, NF-κB, and JAK/STAT signaling pathways are critical in mediating inflammatory responses during cerebral ischemia-reperfusion injury (He et al., 2024; Nazarinia et al., 2021; Zhao et al., 2022; Zhu et al., 2021). In recent years, it has been found that DUBs have an important impact on the activation and inhibition of some important signaling pathways.

NLRP6, a recently identified member of the NLP family, exacerbates neuronal damage following oxygen-glucose deprivation through inflammasome activation and apoptosis induction (Wang et al., 2015; Zhang et al., 2020). This pathway is significantly affected by DUBs during ischemia-reperfusion. For example, BRCC3 regulates multiple cellular processes, including cell cycle control, DNA repair, and immune responses (Sun et al., 2016; Wang et al., 2019). In the context of CIRI, an augmentation in BRCC3 expression was observed, which concomitantly exhibited co-localization with NLRP6 (Huang X. et al., 2021). Concurrently, the BRCC3 influence on the ubiquitination of NLRP6 and the promotion of NLRP6 inflammasome assembly was also evident (Huang et al., 2024).

DUBs also have been shown to play a pivotal role in the NF-κB-related signaling pathway. NF-κB, functioning as a transcription factor, has the capacity to regulate the expression of a multitude of inflammation-related genes (Lawrence, 2009). Harhaj and Dixit systematically elucidated the regulatory mechanisms of A20 and CYLD on NF-κB signaling (Harhaj and Dixit, 2012). As an OTU-family member, A20 (TNFAIP3) employs its distinctive OTU domain to cleave K63-linked ubiquitin chains while utilizing zinc finger domains (ZnF4-7) to catalyze K48-linked polyubiquitination, thereby facilitating proteasomal degradation of key signaling proteins including TNF receptor-associated factor 6 (TRAF6) and receptor-interacting protein kinase 1 (RIP1; Wertz et al., 2004). This dual enzymatic activity establishes a negative feedback loop for NF-κB signaling (Wertz et al., 2004)—a mechanism corroborated by sustained NF-κB activation and pronounced inflammatory phenotypes in A20-knockout mice (Lee et al., 2000). Zhao and colleagues further demonstrated that mesencephalic astrocyte-derived neurotrophic factor promotes microglial polarization toward an anti-inflammatory phenotype via the A20/NF-κB axis, significantly mitigating cerebral ischemia-reperfusion injury in murine models (Zhao et al., 2024). Complementary work by Zhan's group revealed that neuron-specific A20 upregulation effectively suppresses NF-κB-mediated neuroinflammatory damage in rat ischemia-reperfusion models (Zhan et al., 2016). Collectively, these studies establish A20 as a master regulator of neuroinflammatory responses. CYLD, negatively regulates NF-κB signaling by removing K63-linked polyubiquitin chains from key mediators including TNF receptor-associated factors 2 and 6 (TRAF2/6; Trompouki et al., 2003). Emerging evidence demonstrates that CYLD upregulation attenuates inflammatory damage in CIRI through dual mechanisms: (1) direct suppression of NF-κB transcriptional activity (Jiang et al., 2017), and (2) inhibition of NLRP3 inflammasome activation, thereby preventing pathological microglial overactivation and subsequent neuroinflammatory injury in the ischemic cortex (Lin et al., 2021).

OTU domain-containing protein 1 (OTUD1), a crucial member of the OTU protease family, plays a pivotal role in modulating the ubiquitination status of receptor-interacting serine/threonine-protein kinase 2 (RIPK2/RIP2). As a downstream effector of both nucleotide-binding oligomerization domain-containing proteins 1/2 and Toll-like receptor (TLR) signaling pathways (Honjo et al., 2021; Liu et al., 2015), RIP2 undergoes ubiquitination that potently enhances NF-κB-mediated inflammatory signaling (Annibaldi and Meier, 2018). Mechanistic studies demonstrate that during CIRI, OTUD1 directly interacts with either the kinase domain or caspase activation and recruitment domain of RIP2, selectively cleaving its K63-linked polyubiquitin chains. This deubiquitination event effectively suppresses NF-κB pathway activation, thereby attenuating neuroinflammation and ischemic brain damage. Importantly, genetic ablation of OTUD1 exacerbates RIP2 ubiquitination, leading to aggravated cerebral ischemic injury (Zheng et al., 2023). These findings establish the OTUD1-RIP2 axis as a critical regulatory mechanism in CIRI pathogenesis.

TAK1-binding protein 2 (TAB2), a pivotal scaffold protein, orchestrates innate immune responses, inflammatory signaling, and cellular stress reactions by facilitating the assembly of the TGF-β-activated kinase 1 (TAK1) complex, thereby precisely controlling NF-κB pathway activation (Wang et al., 2001; Takaesu et al., 2000). Recent studies demonstrate that the USP25 exerts anti-inflammatory effects during stroke by selectively cleaving K63-linked ubiquitin chains from TAB2, consequently attenuating NF-κB activation and mitigating microglial inflammation (Li Z. et al., 2023).

Furthermore, a pivotal study has elucidated the dual regulatory mechanism of USP8 in neuroinflammatory modulation: in LPS-induced neuroinflammation models, USP8 suppresses the TLR4/MyD88/NF-κB signaling cascade, concurrently inhibiting pro-inflammatory (M1) microglial activation while promoting anti-inflammatory (M2) polarization (Zhao et al., 2020). Functional validation in murine models shows that USP8 overexpression rescues LPS-induced cognitive deficits and motor coordination impairment, whereas USP8 depletion exacerbates neuroinflammatory damage (Zhao et al., 2020). These discoveries not only elucidate USP8's master regulatory role in neuroinflammation but also identify novel therapeutic targets for neurodegenerative disorders.

While USP22 expression is upregulated following CIRI, its biological functions and underlying mechanisms remain incompletely characterized. A groundbreaking study reveals that bone marrow mesenchymal stem cell-derived exosomes deliver long non-coding RNA KLF3-AS1, which functions as a ceRNA to sequester miR-206, thereby relieving miR-206-mediated post-transcriptional repression of USP22 (Xie et al., 2023). The upregulated USP22 in turn stabilizes sirtuin 1 (SIRT1) protein levels through deubiquitination—this NAD^+^-dependent deacetylase executes multifaceted neuroprotective effects, including anti-inflammatory, antioxidant, and anti-apoptotic activities, ultimately improving CIRI outcomes (Xie et al., 2023). This work establishes the KLF3-AS1/miR-206/USP22/SIRT1 axis as a previously unrecognized regulatory pathway in ischemic brain injury.

Emerging evidence indicates that USP14 exerts detrimental effects in ischemic brain injury. A seminal study by Hou et al. (2023) delineates the dual neuroprotective mechanisms of USP14 inhibition in CIRI: (i) At the vascular level, it stabilizes tight junction proteins (occludin and claudin-5) to maintain blood-brain barrier integrity, effectively mitigating vasogenic edema; (ii) In inflammatory modulation, it suppresses microglial hyperactivation and the NF-κB signaling pathway, significantly reducing the release of pro-inflammatory cytokines including TNF-α and IL-6. These findings validate USP14-specific inhibitors (e.g., IU1) as promising therapeutic agents for IS. Notably, follow-up research reveals that microRNA-124 (miR-124) binds the 3′-untranslated region (3′-UTR) of Usp14 mRNA to suppress its expression, thereby attenuating USP14-mediated deubiquitination of RE-1 silencing transcription factor (REST). This promotes REST degradation via the ubiquitin-proteasome system, ultimately relieving REST-mediated transcriptional repression of neuroprotective genes. In focal cerebral ischemia models, this miR-124/Usp14/REST axis demonstrates remarkable neuroprotection, characterized by reduced infarct volume and improved neurological function (Doeppner et al., 2013). This work not only elucidates a novel regulatory axis in ischemic stroke pathogenesis but also provides a theoretical foundation for developing miRNA-based neuroprotective strategies.

Convergent studies have established the pivotal role of ubiquitin C-terminal hydrolase L1 (UCHL1) in axonal preservation and functional recovery following cerebral ischemia, though distinct research groups have elucidated complementary facets of its activity. The Liu group demonstrated that UCHL1 maintains axonal integrity and synaptic plasticity through deubiquitination of structural proteins (e.g., neurofilaments) and synaptic scaffolds (e.g., postsynaptic density protein 95, PSD-95)—genetic ablation exacerbates post-ischemic axonal degeneration and motor deficits (Liu et al., 2019). Complementarily, the Mi group identified the hydrolase activity of UCHL1 as indispensable for neuroprotection: the C90S active-site mutation disrupts ubiquitin homeostasis, precipitating mitochondrial dysfunction and impaired axonal transport, thereby aggravating ischemic injury—a finding that underscores the therapeutic relevance of targeting UCHL1's enzymatic function (Mi et al., 2024). Collectively, UCHL1 orchestrates dual neuroprotective roles in CIRI by modulating the ubiquitin-proteasome system: (1) structural preservation of axonal architecture and (2) functional restoration of neural circuits. These insights posit UCHL1 activators as promising candidates for IS intervention. Notably, whether deubiquitinases engage the JAK/STAT signaling pathway to regulate inflammatory responses in CIRI remains an open question, warranting systematic investigation.

Collectively, these findings position DUBs as master regulators of neuroinflammatory and neuroprotective responses in CIRI, operating through: (i) direct ubiquitin code editing of inflammasome components (BRCC3-NLRP6, CYLD-NLRP3), (ii) multi-layered control of NF-κB signaling (A20/TRAF6, CYLD-TRAF2/6, OTUD1/RIP2, USP25/TAB2), (iii) dynamic modulation of microglial polarization states (USP8-TLR4, USP14-REST), and critically, (iv) axonal integrity preservation via UCHL1-mediated stabilization of synaptic scaffolds (PSD-95/neurofilaments), offering a compelling therapeutic paradigm for targeted immunomodulation in IS. While DUBs modulate inflammatory responses and neuroprotection, their impact on oxidative stress is equally critical, as discussed below.

### 4.2 Oxidative stress modulation

In addition to the inflammatory responses and neuroprotection, DUBs also have been demonstrated to play a very important role in the oxidative stress response after ischemia-reperfusion injury. In IS, heme oxygenase-1 (HO-1) has been found to be highly expressed in the injured brain area, indicating the activation of anti-oxidative stress (Beschorner et al., 2000). Previous studies have shown that KEAP1-NRF2-antioxidant response element (ARE) signaling pathway also plays a very important role in regulating oxidative stress. Under physiological conditions, KEAP1 forms ubiquitin E3 ligase complexes (KEAP1-CUL3) with CULLIN3 (CUL3) and makes NRF2 poly-ubiquitinization (K48-linked), resulting in the rapid degradation of NRF2 by the proteasome system. Under oxidative stress (e.g., CIRI), ROS modify critical cysteine residues in KEAP1 (e.g., Cys151), disrupting the KEAP1-CUL3 complex and inhibiting NRF2 ubiquitination. This stabilizes NRF2, allowing its nuclear translocation to activate antioxidant genes (e.g., HO-1, NQO1; Chan et al., 2021; Rao et al., 2022; Zhao et al., 2018; Lu et al., 2016; Yamamoto et al., 2018). In this process, the study by Tong et al. (2023) reveals that USP16 specifically recognizes the double-glycine repeat domain of KEAP1, selectively cleaving its K48-linked ubiquitin chains to prevent proteasomal degradation and consequently enhance KEAP1 protein stability. Genetic ablation of USP16 significantly ameliorates ischemia-reperfusion-induced cellular damage and inflammatory responses. Mechanistically, nuclear-translocated NRF2 directly binds to the USP16 promoter, establishing a KEAP1-NRF2-USP16 negative feedback loop that refines our understanding of the KEAP1-NRF2-ARE signaling network. In a parallel regulatory mechanism, Mao et al. (2024) demonstrate that USP4 similarly modulates KEAP1 deubiquitination: USP4 overexpression reduces KEAP1 ubiquitination, whereas USP4 knockdown promotes KEAP1 ubiquitination and subsequent degradation. Importantly, pharmacological inhibition of USP4 activity enhances KEAP1 ubiquitination, triggering NRF2-dependent antioxidant responses and conferring robust neuroprotection in stroke models—evidenced by reduced neuronal apoptosis and improved functional recovery.

Accumulating evidence highlights the pivotal role of DUBs in modulating oxidative stress responses in diverse tissues. In hepatic ischemia-reperfusion injury, CYLD physically interacts with NADPH oxidase 4 (NOX4) and enhances NRF2 signaling through NOX4 deubiquitination, thereby mitigating stress-induced hepatic inflammation and programmed cell death (Zhan et al., 2022). Parallel studies demonstrate that USP25 directly binds KEAP1 and impedes its ubiquitin-dependent degradation (Cai et al., 2023), mirroring the regulatory mechanism employed by USP16.

Furthermore, structural analyses reveal that OTUD1 orchestrates cellular responses to oxidative stress and apoptosis via its dedicated KEAP1-binding domain in a ROS-sensitive manner. Of particular note, USP13 overexpression in osteoarthritis models exerts dual antioxidant effects by simultaneously stabilizing NRF2 protein levels and suppressing caspase-3 activation, resulting in markedly reduced ROS accumulation (Huang J. et al., 2021).

Collectively, the identification of the USP16/KEAP1 and USP4/KEAP1 regulatory axes demonstrates that targeted inhibition of specific DUBs can potentiate the NRF2-mediated endogenous antioxidant defense system. This discovery suggests that selective USP16/USP4 inhibitors may synergize with existing ROS scavengers to develop novel combinatorial therapeutic strategies for stroke. Importantly, the spatiotemporal regulation of distinct DUBs in stroke-associated oxidative stress requires systematic elucidation to inform the development of precision therapeutic interventions.

### 4.3 Programmed cell death control

Cerebral ischemia-reperfusion triggers extensive neuronal death, resulting in neurological dysfunction and cognitive impairment following vascular recanalization (Feigin et al., 2017). Central to this pathological cascade is PCD-a process wherein cells actively initiate self-elimination through tightly regulated signaling pathways (e.g., apoptosis, necroptosis, autophagy, pyroptosis, and ferroptosis) upon specific stimuli. Crucially, these PCD modalities exhibit dual regulatory roles in CIRI: while their controlled activation clears damaged cells and mitigates secondary necrosis/inflammation, dysregulated execution exacerbates neuronal injury and functional deficits (Lu et al., 2021; Mashimo et al., 2019; Yang et al., 2014, 2017; Zille et al., 2017).

Recent studies have established that DUBs critically modulate PCD pathways in stroke pathophysiology. The anti-inflammatory protein A20 confers cerebral ischemic tolerance by selectively removing K63-linked ubiquitination from receptor-interacting protein kinase 3 (RIP3), thereby suppressing necroptotic signaling and bidirectionally regulating microglial/macrophage polarization—inhibiting pro-inflammatory M1 phenotype while promoting anti-inflammatory M2 transition (Onizawa et al., 2015; Qiu et al., 2024). These findings not only elucidate the pivotal role of the A20-RIP3 axis in neuroinflammation and cell death but also propose novel therapeutic strategies targeting necroptosis and immune microenvironment remodeling.

In mitochondrial quality control, the mitochondria-localized USP30 maintains network integrity by stabilizing key proteins like MFN2 through deubiquitination. During CIRI, USP30 prevents excessive mitochondrial fission and fragmentation by inhibiting ubiquitin-mediated MFN2 degradation. Functional studies demonstrate that USP30 overexpression preserves mitochondrial function and reduces neuronal apoptosis, whereas its knockout exacerbates injury (Chen et al., 2021), positioning the USP30-MFN2 axis as a promising target for mitochondrial therapy.

Beyond its established anti-inflammatory and antioxidant roles, USP22 orchestrates autophagy through the newly identified USP22-PTEN-mTOR-transcription factor EB (TFEB) axis: USP22 inhibition accelerates PTEN degradation, relieving its suppression on mammalian target of rapamycin complex 1 (mTORC1), which subsequently activates TFEB to enhance lysosomal biogenesis and autophagic flux, ultimately improving neurological recovery (Li Y. et al., 2023). Parallelly, OTUB1 maintains metabolic homeostasis by stabilizing DEPTOR, an endogenous mTORC1 inhibitor. Intriguingly, pathogens like Salmonella hijack host OTUB1 (via bacterial E3 ligase SopA) to manipulate the DEPTOR-mTORC1 axis for autophagy induction and immune evasion (Ma et al., 2024)—though whether this pathway mediates autophagy in CIRI awaits investigation.

Emerging research establishes DUBs as pivotal modulators of ferroptosis. The Li group demonstrated that in ischemic stroke models, nuclear receptor coactivator 4 (NCOA4) drives ferroptosis by mediating ferritinophagy—a selective autophagic process that degrades ferritin, leading to free iron release and lipid peroxidation (Li et al., 2021). Crucially, USP14 directly interacts with NCOA4 and stabilizes it through deubiquitination, thereby amplifying NCOA4-mediated ferritinophagy and exacerbating ferroptotic neuronal death. Pharmacological inhibition of USP14 reduces NCOA4 protein levels, significantly attenuating both ferroptosis and cerebral ischemic injury (Li et al., 2021). This work not only delineates the NCOA4-ferritinophagy-ferroptosis axis but also identifies the USP14-NCOA4 interaction as a novel therapeutic target for stroke. Remarkably, this regulatory paradigm exhibits cross-organ conservation: in renal ischemia-reperfusion injury, USP14 inhibition markedly decreases ROS-dependent ferroptosis in HK-2 cells (Pan et al., 2023), while in cardiac models, OTUD5 upregulation diminishes ubiquitination of glutathione peroxidase 4, preventing its degradation and ultimately reversing 4-HNE-induced ferroptosis to mitigate myocardial damage (Liu et al., 2023).

Collectively, DUBs orchestrate a multi-layered defense against programmed neuronal death in CIRI by: (i) intercepting necroptosis (A20-RIP3), (ii) preserving mitochondrial integrity (USP30-MFN2), (iii) modulating autophagic flux (USP22-PTEN/TFEB), and (iv) suppressing ferroptosis (USP14-NCOA4)—positioning targeted DUB modulation as a strategic approach to concurrently address multiple cell death pathways in IS therapy. Table 1 summarizes the key DUBs, substrates, and molecular mechanisms in CIRI.

**Table 1:** Key DUBs in CIRI and their molecular mechanisms.

**DUBs**	**Families**	**Substrate**	**Role in CIRI**	**References**
BRCC3	JAMM	NLRP6	BRCC3-mediated regulation of NLRP6 inflammasome assembly through ubiquitination modulation	Huang X. et al., 2021; Huang et al., 2024
A20	OUT	TRAF6, RIP1	A20 promotes the degradation of TRAF6 and RIP1, exerting a negative feedback effect on the NF-κB signaling pathway, thereby alleviating inflammatory injury in CIRI	Wertz et al., 2004; Lee et al., 2000; Zhao et al., 2024; Zhan et al., 2016
CYLD	USP	TRAF2, TRAF6, NLRP3	CYLD inhibits the NF-κB signaling pathway and NLRP3 activation, thereby alleviating inflammatory injury in CIRI	Trompouki et al., 2003; Jiang et al., 2017; Lin et al., 2021
OTUD1	OUT	RIP2	OTUD1 suppresses RIP2-induced NF-κB activation by cleaving K63-linked ubiquitin chains on RIP2, thereby mitigating CIRI-induced inflammation and injury	Honjo et al., 2021; Liu et al., 2015; Annibaldi and Meier, 2018; Zheng et al., 2023
USP25	USP	TAB2	USP25 exerts anti-inflammatory effects via selective cleavage of K63-linked ubiquitin chains from TAB2 to attenuate NF-κB activation and microglial inflammation	Wang et al., 2001; Takaesu et al., 2000; Li Z. et al., 2023
USP8	USP	TLR4	USP8 mitigates neuroinflammation by suppressing the TLR4/MyD88/NF-κB signaling pathway, thereby reducing pro-inflammatory (M1) microglial activation and promoting anti-inflammatory (M2) phenotypic polarization	Zhao et al., 2020
USP22	USP	SIRT1	USP22 exerts multiple neuroprotective effects, including anti-inflammatory, antioxidant, and anti-apoptotic functions, by stabilizing SIRT1 protein levels through deubiquitination, thereby significantly improving CIRI outcomes	Xie et al., 2023
USP14	USP	REST; occluding, claudin-5	USP14 stabilizes REST protein and suppresses neuroprotective gene expression. The inhibition of USP14 improves CIRI through dual neuroprotective mechanisms: (1) maintaining blood-brain barrier integrity by stabilizing tight junction proteins (occludin and claudin-5), effectively reducing vasogenic brain edema; and (2) significantly suppressing microglial overactivation and the NF-κB signaling pathway, thereby decreasing the release of pro-inflammatory factors such as TNF-α and IL-6	Hou et al., 2023; Doeppner et al., 2013
UCHL1	UCH	Neurofilament, PSD-95	UCHL1 exerts dual neuroprotective effects in CIRI, promoting both axonal protection and neural functional recovery through modulation of the ubiquitin-proteasome system	Liu et al., 2019; Mi et al., 2024
USP16	USP	KEAP1	USP16 stabilizes KEAP1 Through k48-linked deubiquitination to suppress NRF2-mediated antioxidant responses	Tong et al., 2023
USP4	USP	KEAP1	USP4 exerts pro-oxidative effects through KEAP1 deubiquitination and stabilization	Mao et al., 2024
A20	OUT	RIP3	A20 exerts anti-cell death effects by removing K63-linked ubiquitination of RIP3, thereby blocking the necroptosis signaling pathway	Onizawa et al., 2015; Qiu et al., 2024
USP30	USP	MFN2	USP30 effectively reduces mitochondrial hyper-fission and fragmentation by inhibiting the ubiquitination-mediated degradation of MFN2, thereby attenuating neuronal apoptosis	Chen et al., 2021
USP22	USP	PTEN	Inhibition of USP22 promotes PTEN degradation, thereby relieving its suppression on mTORC1 targets and subsequently activating TFEB to enhance lysosomal biogenesis and autophagic flux, ultimately improving neurological functional recovery	Li Y. et al., 2023
USP14	USP	NCOA4	USP14 stabilizes NCOA4 protein through deubiquitination, enhancing its ferritinophagy activity, which consequently exacerbates ferroptosis and aggravates cerebral ischemic injury	Li et al., 2021

## 5 The therapeutic potential of DUBs in CIRI

Emerging evidence positions DUBs as promising therapeutic targets for CIRI, with their modulation demonstrating significant neuroprotective effects in preclinical models. The dynamic interplay between DUBs and their substrates offers multiple intervention strategies, including small-molecule inhibitors, genetic regulation, and upstream signaling pathway modulation, which collectively address key pathological axes such as inflammation, oxidative stress, and PCD.

### 5.1 Pharmacological inhibition of DUBs

Targeted inhibition of pro-injury DUBs has shown efficacy in reducing neuronal damage. For instance, the USP14-specific inhibitor IU1 suppresses ferroptosis by blocking USP14-mediated stabilization of NCOA4, thereby attenuating ferritinophagy and lipid peroxidation in ischemic brain tissue (Li et al., 2021). Similarly, Vialinin A, a natural compound, inhibits USP4 activity, preventing KEAP1 deubiquitination and enhancing NRF2-driven antioxidant responses, which significantly reduces infarct volume and improves neurological recovery (Mao et al., 2024). These findings highlight the therapeutic potential of selective DUB inhibitors in mitigating CIRI-associated pathways.

### 5.2 Genetic and epigenetic regulation

Genetic silencing or overexpression of DUBs provides further mechanistic insights. siRNA-mediated BRCC3 knockdown disrupts NLRP6 inflammasome activation, suppressing neuroinflammation and neuronal apoptosis in rodent CIRI models (Huang et al., 2024). Conversely, A20 upregulation via MALT1 inhibitors (e.g., MI-2) stabilizes RIPK3 ubiquitination, inhibiting necroptosis and microglial M1 polarization (Qiu et al., 2024). Epigenetic approaches, such as miRNA-124 delivery, downregulate USP14 expression, alleviating REST-mediated repression of neuroprotective genes (Doeppner et al., 2013). These strategies underscore the versatility of DUBs modulation in restoring cellular homeostasis.

### 5.3 Clinical translation challenges

Despite preclinical success, clinical translation faces hurdles. Most studies rely on rodent models, and human DUB isoforms may exhibit divergent substrate specificities or regulatory mechanisms. Additionally, systemic DUBs inhibition risks off-target effects due to their pleiotropic roles. Emerging solutions include tissue-specific delivery systems (e.g., exosomal lncRNAs) and isoform-selective inhibitors, as exemplified by the neuroprotective KLF3-AS1/miR-206/USP22/SIRT1 axis delivered via mesenchymal stem cell exosomes (Xie et al., 2023). Future efforts should prioritize optimizing pharmacokinetics and safety profiles of DUBs-targeted agents.

## 6 Prospects and limitations

The exploration of DUBs in CIRI reveals both opportunities and challenges.

### 6.1 Mechanistic complexity

DUBs operate within intricate networks, where their activity is spatiotemporally regulated by post-translational modifications, subcellular localization, and interacting partners. For example, USP30's mitochondrial anchoring is critical for its anti-fission function, yet ischemia-induced displacement disrupts this protective role (Chen et al., 2021). Similarly, redox-sensitive DUBs like A20 and CYLD exhibit context-dependent roles—neuroprotective in CIRI but potentially oncogenic in other settings (Kulathu et al., 2013). Such complexity necessitates precise targeting strategies to avoid unintended consequences.

### 6.2 Technical and translational barriers

Current limitations include: (i) model discrepancies: most findings derive from transient ischemia models, which inadequately mimic chronic human stroke pathologies. (ii) Ubiquitin code specificity: the diversity of ubiquitin chain linkages (e.g., K48 vs. K63) complicates therapeutic targeting. For instance, OTUD1's selective cleavage of RIP2 K63-linked suppresses inflammation (Zheng et al., 2023), but indiscriminate DUBs activation might disrupt proteostasis. (iii) Clinical validation: No DUBs-targeted therapies have entered stroke clinical trials. Promising candidates like IU1 require rigorous evaluation for blood-brain barrier penetration and long-term safety.

### 6.3 Future directions

(i) Multi-omics profiling: integrate proteomics, ubiquitinomics, and single-cell sequencing to map DUBs-substrate interactions in human stroke tissues. (ii) Nanotechnology-driven delivery: develop nanoparticle carriers for brain-specific DUBs inhibitor delivery, leveraging advances in exosome engineering (Song et al., 2019). (iii) Combination therapies: pair DUBs modulation with reperfusion therapies (e.g., thrombectomy) or antioxidants to amplify neuroprotection. For example, USP16 inhibition synergizes with NRF2 activators to enhance oxidative stress defense (Tong et al., 2023).

## 7 Conclusion

DUBs exhibit dualistic and context-dependent roles in CIRI. Key enzymes such as A20, USP30, and OTUD1 confer neuroprotection by suppressing NF-κB-driven inflammation, preserving mitochondrial integrity, and blocking ferroptosis, whereas aberrant activation of BRCC3 or USP14 exacerbates injury. This functional dichotomy hinges on substrate selectivity, ubiquitin chain-type specificity, and spatiotemporal regulation within ischemic microenvironments. Despite preclinical success in mitigating CIRI through DUB targeting, translational hurdles remain: (i) discrepancies between animal models and human pathophysiology limit mechanistic extrapolation; (ii) the pleiotropic nature of DUB-substrate networks raises concerns about off-target effects; and (iii) blood-brain barrier penetration and long-term safety require rigorous validation. Future efforts should prioritize: (i) mapping DUB-substrate interactions in human ischemic tissues using single-cell sequencing and ubiquitinomics; (ii) engineering nanoparticle or stem cell-derived exosome carriers for brain-specific DUB modulator delivery; and (iii) investigating combinatorial therapies integrating DUB inhibition with thrombolysis or thrombectomy. Through interdisciplinary innovation, DUB-targeted interventions hold transformative potential for post-stroke neurorestoration.

## References

[B1] Amerik A. Y. Hochstrasser M. (2004). Mechanism and function of deubiquitinating enzymes. Biochim. Biophys. Acta 1695, 189–207. 10.1016/j.bbamcr.2004.10.003 15571815

[B2] Annibaldi A. Meier P. (2018). Checkpoints in TNF-induced cell death: implications in inflammation and cancer. Trends Mol. Med. 24, 49–65. 10.1016/j.molmed.2017.11.002 29217118

[B3] Balut C. M. Loch C. M. Devor D. C. (2011). Role of ubiquitylation and USP8-dependent deubiquitylation in the endocytosis and lysosomal targeting of plasma membrane KCa3.1. FASEB J. 25, 3938–3948. 10.1096/fj.11-187005 21828287 PMC3205838

[B4] Berlin I. Schwartz H. Nash P. D. (2010). Regulation of epidermal growth factor receptor ubiquitination and trafficking by the USP8·STAM complex. J. Biol. Chem. 285, 34909–34921. 10.1074/jbc.M109.016287 20736164 PMC2966105

[B5] Beschorner R. Adjodah D. Schwab J. M. Mittelbronn M. Pedal I. Mattern R.. (2000). Long-term expression of heme oxygenase-1 (HO-1, HSP-32) following focal cerebral infarctions and traumatic brain injury in humans. Acta Neuropathol. 100, 377–384. 10.1007/s004010000202 10985695

[B6] Bingol B. Tea J. S. Phu L. Reichelt M. Bakalarski C. E. Song Q.. (2014). The mitochondrial deubiquitinase USP30 opposes parkin-mediated mitophagy. Nature 510, 370–375. 10.1038/nature13418 24896179

[B7] Cai C. Ma H. Peng J. Shen X. Zhen X. Yu C.. (2023). USP25 regulates KEAP1-NRF2 anti-oxidation axis and its inactivation protects acetaminophen-induced liver injury in male mice. Nat. Commun. 14:3648. 10.1038/s41467-023-39412-6 37339955 PMC10282087

[B8] Campbell B. C. V. Khatri P. (2020). Stroke. Lancet 396, 129–142. 10.1016/S0140-6736(20)31179-X 32653056

[B9] Chan B. K. Y. Elmasry M. Forootan S. S. Russomanno G. Bunday T. M. Zhang F.. (2021). Pharmacological activation of Nrf2 enhances functional liver regeneration. Hepatology. 74, 973–986. 10.1002/hep.31859 33872408

[B10] Chen C. Qin H. Tan J. Hu Z. Zeng L. (2020). The role of ubiquitin-proteasome pathway and autophagy-lysosome pathway in cerebral ischemia. Oxid. Med. Cell. Longev. 2020:5457049. 10.1155/2020/5457049 32089771 PMC7016479

[B11] Chen C. Qin H. Tang J. Hu Z. Tan J. Zeng L.. (2021). USP30 protects against oxygen-glucose deprivation/reperfusion induced mitochondrial fragmentation and ubiquitination and degradation of MFN2. Aging 13, 6194–6204. 10.18632/aging.202629 33609088 PMC7950302

[B12] Cheng J. Fan Y. Q. Jiang H. X. Chen S. F. Chen J. Liao X. Y.. (2021). Transcranial direct-current stimulation protects against cerebral ischemia-reperfusion injury through regulating Cezanne-dependent signaling. Exp. Neurol. 345:113818. 10.1016/j.expneurol.2021.113818 34324860

[B13] Cheng J. Guggino W. (2013). Ubiquitination and degradation of CFTR by the E3 ubiquitin ligase MARCH2 through its association with adaptor proteins CAL and STX6. PLoS ONE 8:e68001. 10.1371/journal.pone.0068001 23818989 PMC3688601

[B14] Clague M. J. (2013). Biochemistry: oxidation controls the DUB step. Nature 497, 49–50. 10.1038/497049a 23636394

[B15] Clague M. J. Urbé S. (2006). Endocytosis: the DUB version. Trends Cell Biol. 16, 551–559. 10.1016/j.tcb.2006.09.002 16996268

[B16] Clague M. J. Urbé S. (2017). Integration of cellular ubiquitin and membrane traffic systems: focus on deubiquitylases. FEBS J. 284, 1753–1766. 10.1111/febs.14007 28064438 PMC5484354

[B17] Cohn M. A. Kee Y. Haas W. Gygi S. P. D'Andrea A. D. (2009). UAF1 is a subunit of multiple deubiquitinating enzyme complexes. J. Biol. Chem. 284, 5343–5351. 10.1074/jbc.M808430200 19075014 PMC2643494

[B18] Cotto-Rios X. M. Békés M. Chapman J. Ueberheide B. Huang T. T. (2012). Deubiquitinases as a signaling target of oxidative stress. Cell Rep. 2, 1475–1484. 10.1016/j.celrep.2012.11.011 23219552 PMC3534866

[B19] de Poot S. A. H. Tian G. Finley D. (2017). Meddling with fate: the proteasomal deubiquitinating enzymes. J. Mol. Biol. 429, 3525–3545. 10.1016/j.jmb.2017.09.015 28988953 PMC5675770

[B20] Defelipe L. A. Lanzarotti E. Gauto D. Marti M. A. Turjanski A. G. (2015). Protein topology determines cysteine oxidation fate: the case of sulfenyl amide formation among protein families. PLoS Comput. Biol. 11:e1004051. 10.1371/journal.pcbi.1004051 25741692 PMC4351059

[B21] Devarie-Baez N. O. Silva Lopez E. I. Furdui C. M. (2016). Biological chemistry and functionality of protein sulfenic acids and related thiol modifications. Free Radic. Res. 50, 172–194. 10.3109/10715762.2015.1090571 26340608 PMC5292231

[B22] Doeppner T. R. Doehring M. Bretschneider E. Zechariah A. Kaltwasser B. Müller B.. (2013). MicroRNA-124 protects against focal cerebral ischemia via mechanisms involving Usp14-dependent REST degradation. Acta Neuropathol. 126, 251–265. 10.1007/s00401-013-1142-5 23754622

[B23] Dougherty S. E. Maduka A. O. Inada T. Silva G. M. (2020). Expanding role of ubiquitin in translational control. Int. J. Mol. Sci. 21:1151. 10.3390/ijms21031151 32050486 PMC7037965

[B24] Escobar-Henriques M. (2014). Mitofusins: ubiquitylation promotes fusion. Cell Res. 24, 387–388. 10.1038/cr.2014.23 24556809 PMC3975502

[B25] Feigin V. L. Norrving B. Mensah G. A. (2017). Global burden of stroke. Circ. Res. 120, 439–448. 10.1161/CIRCRESAHA.116.308413 28154096

[B26] Foot N. Henshall T. Kumar S. (2017). Ubiquitination and the regulation of membrane proteins. Physiol. Rev. 97, 253–281. 10.1152/physrev.00012.2016 27932395

[B27] Forman H. J. Davies M. J. Krämer A. C. Miotto G. Zaccarin M. Zhang H.. (2017). Protein cysteine oxidation in redox signaling: caveats on sulfenic acid detection and quantification. Arch. Biochem. Biophys. 617, 26–37. 10.1016/j.abb.2016.09.013 27693037 PMC5318241

[B28] Fujita H. Iwabu Y. Tokunaga K. Tanaka Y. (2013). Membrane-associated RING-CH (MARCH) 8 mediates the ubiquitination and lysosomal degradation of the transferrin receptor. J. Cell Sci. 126(Pt 13), 2798–2809. 10.1242/jcs.119909 23606747

[B29] Gersch M. Gladkova C. Schubert A. F. Michel M. A. Maslen S. Komander D.. (2017). Mechanism and regulation of the Lys6-selective deubiquitinase USP30. Nat. Struct. Mol. Biol. 24, 920–930. 10.1038/nsmb.3475 28945249 PMC5757785

[B30] Guo N. J. Wang B. Zhang Y. Kang H. Q. Nie H. Q. Feng M. K.. (2024). USP7 as an emerging therapeutic target: a key regulator of protein homeostasis. Int. J. Biol. Macromol. 263(Pt 1):130309. 10.1016/j.ijbiomac.2024.130309 38382779

[B31] Haglund K. Sigismund S. Polo S. Szymkiewicz I. Di Fiore P. P. Dikic I.. (2003). Multiple monoubiquitination of RTKs is sufficient for their endocytosis and degradation. Nat. Cell Biol. 5, 461–466. 10.1038/ncb983 12717448

[B32] Harhaj E. W. Dixit V. M. (2012). Regulation of NF-κB by deubiquitinases. Immunol. Rev. 246, 107–124. 10.1111/j.1600-065X.2012.01100.x 22435550 PMC3540820

[B33] He Q. Meng C. Jia M. Tan J. Huang K. Gan H.. (2024). NLRP6 deficiency inhibits neuroinflammation and ameliorates brain injury in ischemic stroke by blocking NLRs inflammasomes activation through proteasomal degradation of pro-caspase-1. Neurobiol. Dis. 192:106434. 10.1016/j.nbd.2024.106434 38341160

[B34] Hecht N. Schrammel M. Neumann K. Müller M. M. Dreier J. P. Vajkoczy P.. (2021). Perfusion-dependent cerebral autoregulation impairment in hemispheric stroke. Ann. Neurol. 89, 358–368. 10.1002/ana.25963 33219550

[B35] Hershko A. Ciechanover A. (1998). The ubiquitin system. Annu. Rev. Biochem. 67, 425–479. 10.1146/annurev.biochem.67.1.425 9759494

[B36] Hochrainer K. (2018). Protein modifications with ubiquitin as response to cerebral ischemia-reperfusion injury. Transl. Stroke Res. 9, 157–173. 10.1007/s12975-017-0567-x 28842824

[B37] Honjo H. Watanabe T. Kamata K. Minaga K. Kudo M. (2021). RIPK2 as a new therapeutic target in inflammatory bowel diseases. Front. Pharmacol. 12:650403. 10.3389/fphar.2021.650403 33935757 PMC8079979

[B38] Hou W. Yao J. Liu J. Lin X. Wei J. Yin X.. (2023). USP14 inhibition promotes recovery by protecting BBB integrity and attenuating neuroinflammation in MCAO mice. CNS Neurosci. Ther. 29, 3612–3623. 10.1111/cns.14292 37269080 PMC10580339

[B39] Huang J. Ye Z. Wang J. Chen Q. Huang D. Liu H.. (2021). USP13 mediates PTEN to ameliorate osteoarthritis by restraining oxidative stress, apoptosis and inflammation via AKT-dependent manner. Biomed. Pharmacother. 133:111089. 10.1016/j.biopha.2020.111089 33378983

[B40] Huang X. Gan H. Tan J. Wang T. Zhao J. Zhao Y.. (2021). BRCC3 promotes activation of the NLRP6 inflammasome following cerebral ischemia/reperfusion (I/R) injury in rats. Neurosci. Lett. 756:135954. 10.1016/j.neulet.2021.135954 33979701

[B41] Huang X. Tan J. Ji Y. Luo J. Zhao Y. Zhao J.. (2024). BRCC3 mediates inflammation and pyroptosis in cerebral ischemia/reperfusion injury by activating the NLRP6 inflammasome. CNS Neurosci. Ther. 30:e14697. 10.1111/cns.14697 38544474 PMC10973773

[B42] Hurley J. H. Stenmark H. (2011). Molecular mechanisms of ubiquitin-dependent membrane traffic. Annu. Rev. Biophys. 40, 119–142. 10.1146/annurev-biophys-042910-155404 21332354 PMC3272705

[B43] Hutti J. E. Shen R. R. Abbott D. W. Zhou A. Y. Sprott K. M. Asara J. M.. (2009). Phosphorylation of the tumor suppressor CYLD by the breast cancer oncogene IKKepsilon promotes cell transformation. Mol. Cell. 34, 461–472. 10.1016/j.molcel.2009.04.031 19481526 PMC2746958

[B44] Jacomin A. C. Taillebourg E. Fauvarque M. O. (2018). Deubiquitinating enzymes related to autophagy: new therapeutic opportunities? Cells 7:112. 10.3390/cells7080112 30126257 PMC6116007

[B45] Jayaraj R. L. Azimullah S. Beiram R. Jalal F. Y. Rosenberg G. A. (2019). Neuroinflammation: friend and foe for ischemic stroke. J. Neuroinflamm. 16:142. 10.1186/s12974-019-1516-2 31291966 PMC6617684

[B46] Jiang J. Luo Y. Qin W. Ma H. Li Q. Zhan J.. (2017). Electroacupuncture suppresses the NF-κB signaling pathway by upregulating cylindromatosis to alleviate inflammatory injury in cerebral ischemia/reperfusion rats. Front. Mol. Neurosci. 10:363. 10.3389/fnmol.2017.00363 29163038 PMC5681846

[B47] Jing M. Bao L. X. Y. Seet R. C. S. (2023). Estimated incidence and mortality of stroke in China. JAMA Netw Open 6:e231468. 10.1001/jamanetworkopen.2023.1468 36862416

[B48] Kahles T. Poon C. Qian L. Palfini V. Srinivasan S. P. Swaminathan S.. (2021). Elevated post-ischemic ubiquitination results from suppression of deubiquitinase activity and not proteasome inhibition. Cell. Mol. Life Sci. 78, 2169–2183. 10.1007/s00018-020-03625-5 32889561 PMC7933347

[B49] Kapadia B. B. Gartenhaus R. B. (2019). DUBbing down translation: the functional interaction of deubiquitinases with the translational machinery. Mol. Cancer Ther. 18, 1475–1483. 10.1158/1535-7163.MCT-19-0307 31481479 PMC6727985

[B50] Kee Y. Huang T. T. (2016). Role of deubiquitinating enzymes in DNA repair. Mol. Cell. Biol. 36, 524–544. 10.1128/MCB.00847-15 26644404 PMC4751696

[B51] Kee Y. Yang K. Cohn M. A. Haas W. Gygi S. P. D'Andrea A. D.. (2010). WDR20 regulates activity of the USP12 x UAF1 deubiquitinating enzyme complex. J. Biol. Chem. 285, 11252–11257. 10.1074/jbc.M109.095141 20147737 PMC2857003

[B52] Khoronenkova S. V. Dianova I. I. Ternette N. Kessler B. M. Parsons J. L. Dianov G. L.. (2012). ATM-dependent downregulation of USP7/HAUSP by PPM1G activates p53 response to DNA damage. Mol. Cell. 45, 801–813. 10.1016/j.molcel.2012.01.021 22361354 PMC3401373

[B53] Kim W. Bennett E. J. Huttlin E. L. Guo A. Li J. Possemato A.. (2011). Systematic and quantitative assessment of the ubiquitin-modified proteome. Mol. Cell. 44, 325–340. 10.1016/j.molcel.2011.08.025 21906983 PMC3200427

[B54] Komander D. Rape M. (2012). The ubiquitin code. Annu. Rev. Biochem. 81, 203–229. 10.1146/annurev-biochem-060310-170328 22524316

[B55] Kulathu Y. Garcia F. J. Mevissen T. E. Busch M. Arnaudo N. Carroll K. S.. (2013). Regulation of A20 and other OTU deubiquitinases by reversible oxidation. Nat. Commun. 4:1569. 10.1038/ncomms2567 23463012 PMC4176832

[B56] Lawrence T. (2009). The nuclear factor NF-kappaB pathway in inflammation. Cold Spring Harb. Perspect. Biol. 1:a001651. 10.1101/cshperspect.a001651 20457564 PMC2882124

[B57] Lee E. G. Boone D. L. Chai S. Libby S. L. Chien M. Lodolce J. P.. (2000). Failure to regulate TNF-induced NF-kappaB and cell death responses in A20-deficient mice. Science 289, 2350–2354. 10.1126/science.289.5488.2350 11009421 PMC3582399

[B58] Lee J. G. Baek K. Soetandyo N. Ye Y. (2013). Reversible inactivation of deubiquitinases by reactive oxygen species in vitro and in cells. Nat. Commun. 4:1568. 10.1038/ncomms2532 23463011 PMC3615374

[B59] Li C. Sun G. Chen B. Xu L. Ye Y. He J.. (2021). Nuclear receptor coactivator 4-mediated ferritinophagy contributes to cerebral ischemia-induced ferroptosis in ischemic stroke. Pharmacol Res. 174:105933. 10.1016/j.phrs.2021.105933 34634471

[B60] Li Y. Gao J. Liu C. Bu N. Zhan S. Wu H.. (2023). USP22 knockdown protects against cerebral ischemia/reperfusion injury via destabilizing PTEN protein and activating the mTOR/TFEB pathway. Naunyn Schmiedebergs. Arch. Pharmacol. 396, 3163–3175. 10.1007/s00210-023-02524-3 37191727

[B61] Li Y. Reverter D. (2021). Molecular Mechanisms of DUBs Regulation in Signaling and Disease. Int. J. Mol. Sci. 22:986. 10.3390/ijms22030986 33498168 PMC7863924

[B62] Li Z. Liu B. Lambertsen K. L. Clausen B. H. Zhu Z. Du X.. (2023). USP25 inhibits neuroinflammatory responses after cerebral ischemic stroke by deubiquitinating TAB2. Adv. Sci. 10:e2301641. 10.1002/advs.202301641 37587766 PMC10558664

[B63] Lin X. Zhan J. Jiang J. Ren Y. (2021). Upregulation of neuronal cylindromatosis expression is essential for electroacupuncture-mediated alleviation of neuroinflammatory injury by regulating microglial polarization in rats subjected to focal cerebral ischemia/reperfusion. J. Inflamm. Res. 14, 2061–2078. 10.2147/JIR.S307841 34045881 PMC8149215

[B64] Liu H. Povysheva N. Rose M. E. Mi Z. Banton J. S. Li W.. (2019). Role of UCHL1 in axonal injury and functional recovery after cerebral ischemia. Proc. Natl. Acad. Sci. U.S.A. 116, 4643–4650. 10.1073/pnas.1821282116 30760601 PMC6410860

[B65] Liu H. Wei X. Kong L. Liu X. Cheng L. Yan S.. (2015). NOD2 is involved in the inflammatory response after cerebral ischemia-reperfusion injury and triggers NADPH oxidase 2-derived reactive oxygen species. Int. J. Biol. Sci. 11, 525–535. 10.7150/ijbs.10927 25892960 PMC4400384

[B66] Liu L. Pang J. Qin D. Li R. Zou D. Chi K.. (2023). Deubiquitinase OTUD5 as a novel protector against 4-HNE-triggered ferroptosis in myocardial ischemia/reperfusion injury. Adv. Sci. 10:e2301852. 10.1002/advs.202301852 37552043 PMC10558642

[B67] Liu X. Yamashita T. Shang J. Shi X. Morihara R. Huang Y.. (2020). Molecular switching from ubiquitin-proteasome to autophagy pathways in mice stroke model. J. Cereb. Blood Flow Metab. 40, 214–224. 10.1177/0271678X18810617 30375939 PMC6928553

[B68] Lu L. Q. Tian J. Luo X. J. Peng J. (2021). Targeting the pathways of regulated necrosis: a potential strategy for alleviation of cardio-cerebrovascular injury. Cell. Mol. Life Sci. 78, 63–78. 10.1007/s00018-020-03587-8 32596778 PMC11072340

[B69] Lu M. C. Ji J. A. Jiang Z. Y. You Q. D. (2016). The Keap1-Nrf2-ARE pathway as a potential preventive and therapeutic target: an update. Med. Res. Rev. 36, 924–963. 10.1002/med.21396 27192495

[B70] Ma K. Xian W. Liu H. Shu R. Ge J. Luo Z. Q.. (2024). Bacterial ubiquitin ligases hijack the host deubiquitinase OTUB1 to inhibit MTORC1 signaling and promote autophagy. Autophagy 20, 1968–1983. 10.1080/15548627.2024.2353492 38818749 PMC11346569

[B71] Mao M. Xia Q. Zhan G. Bing H. Zhang C. Wang J.. (2024). Vialinin A alleviates oxidative stress and neuronal injuries after ischaemic stroke by accelerating Keap1 degradation through inhibiting USP4-mediated deubiquitination. Phytomedicine 124:155304. 10.1016/j.phymed.2023.155304 38176274

[B72] Marcassa E. Kallinos A. Jardine J. Rusilowicz-Jones E. V. Martinez A. Kuehl S.. (2018). Dual role of USP30 in controlling basal pexophagy and mitophagy. EMBO Rep. 19:e45595. 10.15252/embr.201745595 29895712 PMC6030704

[B73] Marques B. L. Carvalho G. A. Freitas E. M. M. Chiareli R. A. Barbosa T. G. Di Araújo A. G. P.. (2019). The role of neurogenesis in neurorepair after ischemic stroke. Semin. Cell Dev. Biol. 95, 98–110. 10.1016/j.semcdb.2018.12.003 30550812

[B74] Mashimo M. Bu X. Aoyama K. Kato J. Ishiwata-Endo H. Stevens L. A.. (2019). PARP1 inhibition alleviates injury in ARH3-deficient mice and human cells. JCI Insight 4:e124519. 10.1172/jci.insight.124519 30830864 PMC6478412

[B75] Mi Z. Povysheva N. Rose M. E. Ma J. Zeh D. J. Harikumar N.. (2024). Abolishing UCHL1's hydrolase activity exacerbates ischemia-induced axonal injury and functional deficits in mice. J. Cereb. Blood Flow Metab. 44, 1349–1361. 10.1177/0271678X241258809 38833565 PMC11542126

[B76] Muratani M. Tansey W. P. (2003). How the ubiquitin-proteasome system controls transcription. Nat. Rev. Mol. Cell Biol. 4, 192–201. 10.1038/nrm1049 12612638

[B77] Nazarinia D. Dolatshahi M. Faezi M. Nasseri Maleki S. Aboutaleb N. (2021). TLR4/NF-κB and JAK2/STAT3 signaling pathways: cellular signaling pathways targeted by cell-conditioned medium therapy in protection against ischemic stroke. J. Chem. Neuroanat. 113:101938. 10.1016/j.jchemneu.2021.101938 33636320

[B78] Onizawa M. Oshima S. Schulze-Topphoff U. Oses-Prieto J. A. Lu T. Tavares R.. (2015). The ubiquitin-modifying enzyme A20 restricts ubiquitination of the kinase RIPK3 and protects cells from necroptosis. Nat. Immunol. 16, 618–627. 10.1038/ni.3172 25939025 PMC4439357

[B79] Pan J. Zhao J. Feng L. Xu X. He Z. Liang W.. (2023). Inhibition of USP14 suppresses ROS-dependent ferroptosis and alleviates renal ischemia/reperfusion injury. Cell Biochem. Biophys. 81, 87–96. 10.1007/s12013-022-01107-y 36255562

[B80] Park C. W. Ryu K. Y. (2014). Cellular ubiquitin pool dynamics and homeostasis. BMB Rep. 47, 475–482. 10.5483/BMBRep.2014.47.9.128 24924398 PMC4206721

[B81] Park J. Cho J. Kim E. E. Song E. J. (2019). Deubiquitinating enzymes: a critical regulator of mitosis. Int. J. Mol. Sci. 20:5997. 10.3390/ijms20235997 31795161 PMC6929034

[B82] Piper R. C. Dikic I. Lukacs G. L. (2014). Ubiquitin-dependent sorting in endocytosis. Cold Spring Harb. Perspect. Biol. 6:a016808. 10.1101/cshperspect.a016808 24384571 PMC3941215

[B83] Qiu M. Zhang W. Dai J. Sun W. Lai M. Tang S.. (2024). A20 negatively regulates necroptosis-induced microglia/macrophages polarization and mediates cerebral ischemic tolerance via inhibiting the ubiquitination of RIP3. Cell Death Dis. 15:904. 10.1038/s41419-024-07293-2 39695113 PMC11655947

[B84] Rao J. Qiu J. Ni M. Wang H. Wang P. Zhang L.. (2022). Macrophage nuclear factor erythroid 2-related factor 2 deficiency promotes innate immune activation by tissue inhibitor of metalloproteinase 3-mediated RhoA/ROCK pathway in the ischemic liver. Hepatology 75, 1429–1445. 10.1002/hep.32184 34624146 PMC9300153

[B85] Sarkari F. La Delfa A. Arrowsmith C. H. Frappier L. Sheng Y. Saridakis V.. (2010). Further insight into substrate recognition by USP7: structural and biochemical analysis of the HdmX and Hdm2 interactions with USP7. J. Mol. Biol. 402, 825–837. 10.1016/j.jmb.2010.08.017 20713061

[B86] Sheng Y. Saridakis V. Sarkari F. Duan S. Wu T. Arrowsmith C. H.. (2006). Molecular recognition of p53 and MDM2 by USP7/HAUSP. Nat. Struct. Mol. Biol. 13, 285–291. 10.1038/nsmb1067 16474402

[B87] Simões V. Cizubu B. K. Harley L. Zhou Y. Pajak J. Snyder N. A.. (2022). Redox-sensitive E2 Rad6 controls cellular response to oxidative stress via K63-linked ubiquitination of ribosomes. Cell Rep. 39:110860. 10.1016/j.celrep.2022.110860 35613580 PMC9215706

[B88] Song Y. Li Z. He T. Qu M. Jiang L. Li W.. (2019). M2 microglia-derived exosomes protect the mouse brain from ischemia-reperfusion injury via exosomal miR-124. Theranostics 9, 2910–2923. 10.7150/thno.30879 31244932 PMC6568171

[B89] Sun Z. Z. Zhang T. Ning K. Zhu R. Liu F. Tang S. C.. (2016). B7-H3 upregulates BRCC3 expression, antagonizing DNA damage caused by 5-Fu. Oncol. Rep. 36, 231–238. 10.3892/or.2016.4808 27175567

[B90] Takaesu G. Kishida S. Hiyama A. Yamaguchi K. Shibuya H. Irie K.. (2000). TAB2, a novel adaptor protein, mediates activation of TAK1 MAPKKK by linking TAK1 to TRAF6 in the IL-1 signal transduction pathway. Mol. Cell. 5, 649–658. 10.1016/S1097-2765(00)80244-0 10882101

[B91] Tong G. Chen Y. Chen X. Fan J. Zhu K. Hu Z.. (2023). FGF18 alleviates hepatic ischemia-reperfusion injury via the USP16-mediated KEAP1/Nrf2 signaling pathway in male mice. Nat. Commun. 14:6107. 10.1038/s41467-023-41800-x 37777507 PMC10542385

[B92] Trompouki E. Hatzivassiliou E. Tsichritzis T. Farmer H. Ashworth A. Mosialos G. C. Y. L. D.. (2003). is a deubiquitinating enzyme that negatively regulates NF-kappaB activation by TNFR family members. Nature 424, 793–796. 10.1038/nature01803 12917689

[B93] Tu W. J. Wang L. D. (2023). China stroke surveillance report 2021. Mil. Med. Res. 10:33. 10.1186/s40779-023-00463-x 37468952 PMC10355019

[B94] Vostal L. E. Dahan N. E. Reynolds M. J. Kronenberg L. I. Kapoor T. M. (2025). Structural insights into the coupling between VCP, an essential unfoldase, and a deubiquitinase. J. Cell Biol. 224:e202410148. 10.1083/jcb.202410148 40085114 PMC11908451

[B95] Wang C. Deng L. Hong M. Akkaraju G. R. Inoue J. Chen Z. J.. (2001). TAK1 is a ubiquitin-dependent kinase of MKK and IKK. Nature 412, 346–351. 10.1038/35085597 11460167

[B96] Wang C. Y. Deneen B. Tzeng S. F. (2019). BRCA1/BRCA2-containing complex subunit 3 controls oligodendrocyte differentiation by dynamically regulating lysine 63-linked ubiquitination. Glia 67, 1775–1792. 10.1002/glia.23660 31184779

[B97] Wang F. Ning S. Yu B. Wang Y. (2021). USP14: structure, function, and target inhibition. Front. Pharmacol. 12:801328. 10.3389/fphar.2021.801328 35069211 PMC8766727

[B98] Wang P. Zhu S. Yang L. Cui S. Pan W. Jackson R.. (2015). Nlrp6 regulates intestinal antiviral innate immunity. Science 350, 826–830. 10.1126/science.aab3145 26494172 PMC4927078

[B99] Wertz I. E. O'Rourke K. M. Zhou H. Eby M. Aravind L. Seshagiri S.. (2004). De-ubiquitination and ubiquitin ligase domains of A20 downregulate NF-kappaB signalling. Nature 430, 694–699. 10.1038/nature02794 15258597

[B100] Xie X. Cao Y. Dai L. Zhou D. (2023). Bone marrow mesenchymal stem cell-derived exosomal lncRNA KLF3-AS1 stabilizes Sirt1 protein to improve cerebral ischemia/reperfusion injury via miR-206/USP22 axis. Mol. Med. 29:3. 10.1186/s10020-022-00595-1 36627572 PMC9830826

[B101] Yamamoto M. Kensler T. W. Motohashi H. (2018). The KEAP1-NRF2 system: a thiol-based sensor-effector apparatus for maintaining redox homeostasis. Physiol. Rev. 98, 1169–1203. 10.1152/physrev.00023.2017 29717933 PMC9762786

[B102] Yang F. Wang Z. Wei X. Han H. Meng X. Zhang Y.. (2014). NLRP3 deficiency ameliorates neurovascular damage in experimental ischemic stroke. J. Cereb. Blood Flow Metab. 34, 660–667. 10.1038/jcbfm.2013.242 24424382 PMC3982086

[B103] Yang R. Hu K. Chen J. Zhu S. Li L. Lu H.. (2017). Necrostatin-1 protects hippocampal neurons against ischemia/reperfusion injury via the RIP3/DAXX signaling pathway in rats. Neurosci. Lett. 651, 207–215. 10.1016/j.neulet.2017.05.016 28501693

[B104] Yin J. Schoeffler A. J. Wickliffe K. Newton K. Starovasnik M. A. Dueber E. C.. (2015). Structural insights into WD-Repeat 48 activation of ubiquitin-specific protease 46. Structure 23, 2043–2054. 10.1016/j.str.2015.08.010 26388029

[B105] Yue W. Chen Z. Liu H. Yan C. Chen M. Feng D.. (2014). A small natural molecule promotes mitochondrial fusion through inhibition of the deubiquitinase USP30. Cell Res. 24, 482–496. 10.1038/cr.2014.20 24513856 PMC3975501

[B106] Zhan J. Qin W. Zhang Y. Jiang J. Ma H. Li Q.. (2016). Upregulation of neuronal zinc finger protein A20 expression is required for electroacupuncture to attenuate the cerebral inflammatory injury mediated by the nuclear factor-kB signaling pathway in cerebral ischemia/reperfusion rats. J. Neuroinflamm. 13:258. 10.1186/s12974-016-0731-3 27716383 PMC5048665

[B107] Zhan Y. Xu D. Tian Y. Qu X. Sheng M. Lin Y.. (2022). Novel role of macrophage TXNIP-mediated CYLD-NRF2-OASL1 axis in stress-induced liver inflammation and cell death. JHEP Rep. 4:100532. 10.1016/j.jhepr.2022.100532 36035360 PMC9404660

[B108] Zhang J. Jiang N. Zhang L. Meng C. Zhao J. Wu J.. (2020). NLRP6 expressed in astrocytes aggravates neurons injury after OGD/R through activating the inflammasome and inducing pyroptosis. Int. Immunopharmacol. 80:106183. 10.1016/j.intimp.2019.106183 31927506

[B109] Zhang Y. Y. Tian J. Peng Z. M. Liu B. Peng Y. W. Zhang X. J.. (2023). Caspofungin suppresses brain cell necroptosis in ischemic stroke rats via up-regulation of Pellino3. Cardiovasc. Drugs Ther. 37, 9–23. 10.1007/s10557-021-07231-w 34495409

[B110] Zhao D. Ji J. Li S. Wu A. (2022). Skullcapflavone II protects neuronal damage in cerebral ischemic rats via inhibiting NF-κB and promoting angiogenesis. Microvasc. Res. 141:104318. 10.1016/j.mvr.2022.104318 35026288

[B111] Zhao J. Bi W. Zhang J. Xiao S. Zhou R. Tsang C. K.. (2020). USP8 protects against lipopolysaccharide-induced cognitive and motor deficits by modulating microglia phenotypes through TLR4/MyD88/NF-κB signaling pathway in mice. Brain Behav. Immun. 88, 582–596. 10.1016/j.bbi.2020.04.052 32335193

[B112] Zhao X. Wang Z. Wang J. Xu F. Zhang Y. Han D.. (2024). Mesencephalic astrocyte-derived neurotrophic factor (MANF) alleviates cerebral ischemia/reperfusion injury in mice by regulating microglia polarization via A20/NF-κB pathway. Int. Immunopharmacol. 127:111396. 10.1016/j.intimp.2023.111396 38134597

[B113] Zhao X. J. Yu H. W. Yang Y. Z. Wu W. Y. Chen T. Y. Jia K. K.. (2018). Polydatin prevents fructose-induced liver inflammation and lipid deposition through increasing miR-200a to regulate Keap1/Nrf2 pathway. Redox Biol. 18, 124–137. 10.1016/j.redox.2018.07.002 30014902 PMC6068203

[B114] Zheng S. Li Y. Song X. Wu M. Yu L. Huang G.. (2023). OTUD1 ameliorates cerebral ischemic injury through inhibiting inflammation by disrupting K63-linked deubiquitination of RIP2. J. Neuroinflamm. 20:281. 10.1186/s12974-023-02968-7 38012669 PMC10680203

[B115] Zhu H. Jian Z. Zhong Y. Ye Y. Zhang Y. Hu X.. (2021). Janus kinase inhibition ameliorates ischemic stroke injury and neuroinflammation through reducing NLRP3 inflammasome activation via JAK2/STAT3 pathway inhibition. Front. Immunol. 12:714943. 10.3389/fimmu.2021.714943 34367186 PMC8339584

[B116] Zille M. Karuppagounder S. S. Chen Y. Gough P. J. Bertin J. Finger J.. (2017). Neuronal death after hemorrhagic stroke *in vitro* and *in vivo* shares features of ferroptosis and necroptosis. Stroke. 48, 1033–1043. 10.1161/STROKEAHA.116.015609 28250197 PMC5613764

